# Sulfonamides Inhibit Root Growth via ROS-Triggered and MPK3/6-Modulated Synthesis of the Ethylene Precursor ACC

**DOI:** 10.3390/biology15151278

**Published:** 2026-08-03

**Authors:** Ting He, Zixuan Zhao, Xinyi Liu, Hongxia Chang, Zhixuan Du, Longfei Zhu, Guanping Feng

**Affiliations:** Key Laboratory of Jiangxi Province for Biological Invasion and Biosecurity, School of Life Sciences, Jinggangshan University, Ji’an 343009, China

**Keywords:** sulfonamides, ethylene precursor ACC, ACS1, ROS, MPK3/6

## Abstract

Sulfonamide antibiotics threaten ecosystems, yet their phytotoxic mechanisms remain elusive. Here, we demonstrate that sulfonamides inhibit root growth by disrupting the ROS-ethylene signaling axis. Sulfadiazine (SD) strongly inhibits root elongation, an effect strictly dependent on ACS1, as the *acs1-1* mutant is completely insensitive. Mechanistically, SD triggers an RBOH-dependent ROS burst that transcriptionally induces *ACS1*. Since ACS1 forms active heterodimers with ACS2/ACS6, *acs2-1* and *acs6-1* mutants exhibit partial insensitivity. Furthermore, SD partially depends on the MPK3/6 cascade to post-translationally stabilize ACS2/ACS6, with *mpk3* and *mpk6* mutants showing partial resistance. Collectively, sulfonamides employ a dual-pronged mechanism: ROS transcriptionally activate *ACS1*, while MPK3/6 post-translationally stabilizes ACS2/ACS6, driving aberrant ethylene precursor ACC biosynthesis and root growth inhibition.

## Simple Summary

Sulfonamide antibiotic pollution poses an emerging ecological threat to plant ecosystems. Although exposure to these compounds has been shown to induce growth retardation and oxidative stress in plants, exactly how they disrupt primary cellular metabolism and trigger downstream stress signaling pathways remains largely elusive. This study investigated how these drugs inhibit root growth. We discovered that sulfonamides interfere with the plant’s natural stress and hormone systems. Plants typically use mild stress signals (ROS) and the hormone ethylene to survive harsh conditions. Instead, sulfonamides trigger a massive false stress signal that activates genes for ethylene production, telling roots to stop growing. Furthermore, the drugs manipulate cellular machinery to lock ethylene production in the “on” position, preventing roots from recovering. In conclusion, sulfonamides launch a two-pronged attack: they use a false stress signal to start ethylene production and disable the natural shut-off switch. For society, this discovery highlights the hidden ecological dangers of antibiotic pollution, providing crucial knowledge to help farmers and environmentalists protect crops and ecosystems.

## 1. Introduction

Sulfonamides are broad-spectrum antimicrobials extensively utilized in human healthcare, livestock, and aquaculture to combat bacterial infections [1,2]. However, due to their inherent chemical stability, high aqueous solubility, and poor biological metabolization—approximately 90% of the ingested dose is excreted in bioactive forms—these agents have become ubiquitous environmental contaminants [3,4]. They routinely infiltrate aquatic systems via pharmaceutical wastewater, agricultural runoff, and aquaculture discharges [5,6]. Consequently, sulfonamide antibiotics are now ubiquitous in aquatic environments, frequently detected at μg/L levels in both surface water and groundwater. Of greater concern, agricultural hotspots suffer from severe local accumulation due to the land application of manure. For instance, investigations in Zhejiang Province have reported sulfonamide concentrations in swine manure reaching up to 46.37 mg/kg, highlighting an intense point-source pollution load that vastly exceeds typical aquatic levels [7,8,9].

Functioning as structural analogs of para-aminobenzoic acid (PABA), sulfonamides competitively inhibit dihydropteroate synthase (DHPS) to disrupt the *de novo* folate synthesis pathway and halt microbial growth [10]. Once present in the environment, chronic exposure to these compounds induces multifaceted ecotoxicological effects on non-target flora and fauna, including metabolic, reproductive, and developmental impairments [11,12,13]. Compounding this direct ecotoxicity, sub-inhibitory environmental concentrations of sulfonamides exert continuous selective pressure, driving the enrichment of sulfonamide-resistant bacteria and the horizontal dissemination of antibiotic resistance genes (ARGs) [14]. The propagation of these ARGs across environmental matrices—and their potential transfer to human pathogens—constitutes a severe public health threat, increasingly framed as a “silent pandemic” within the One Health paradigm [15].

Sulfonamide exposure induces significant phytotoxicity across both terrestrial and aquatic plant species. At the cellular level, these compounds repress cell proliferation in the root apical meristem by competitively inhibiting folate biosynthesis, leading to altered root architecture in terrestrial models (e.g., barley and Arabidopsis) and suppressed germination in crops [16,17]. Beyond this canonical folate-targeting pathway, recent evidence indicates that sulfonamide-mediated growth inhibition is also heavily dependent on the isochorismate synthase (ICS1)-catalyzed biosynthesis of salicylic acid [18]. In aquatic ecosystems, longitudinal risk assessments demonstrate that sulfonamides act as persistent multigenerational stressors on duckweed. Despite environmental attenuation processes, partial elimination of parent compounds fails to mitigate long-term toxicity, resulting in acute growth inhibition and chronic developmental impairments [15,19]. Although research on the effects of sulfonamide exposure on plant growth and development is increasing, studies focusing on the underlying molecular mechanisms remain severely lacking.

To dissect the molecular mechanisms underlying sulfonamide phytotoxicity, we performed a large-scale screen for Arabidopsis mutants resistant to root growth inhibition on sulfonamide-supplemented 1/2MS medium. Notably, we identified a loss-of-function mutant of *ACS1*, which encodes 1-aminocyclopropane-1-carboxylate (ACC) synthase, the rate-limiting enzyme in ethylene biosynthesis, that was completely insensitive to the treatment. This finding demonstrates that the inhibitory effects of sulfonamides fundamentally depend on the ethylene biosynthesis pathway.

## 2. Materials and Methods

### 2.1. Plant Materials and Growth Conditions

In this study, Arabidopsis thaliana ecotype Columbia-0 (Col-0) and relevant mutant lines (*acs1-1*, CS16563; *acs1-2*, SALK_202676C; *acs2-1*, CS16564; *acs6-1*, SAIL_832_D08; *acs5*, CS16651; *rboh-DF*, CS9558; *mpk3*, SALK_151594; *mpk6*, CS31099; *proACS1::GUS*, CS31379) sourced from the Nottingham Arabidopsis Stock Centre (NASC, UK) were utilized. Following surface sterilization, seeds were plated onto 1/2 MS medium supplemented with 1% (*w*/*v*) sucrose and solidified with 0.6% (*w*/*v*) agar. Seeds were germinated, and seedlings cultivated in a controlled chamber maintained at 22 ± 1 °C, with a 16/8 h light/dark cycle, a light intensity of 80–90 µmol m^−2^ s^−1^, and 65% relative humidity.

### 2.2. Cytological Observation and Tissue Staining

For GUS staining, samples were incubated in a solution containing 1 mM X-Gluc, 0.1% Triton X-100, 2 mM potassium ferricyanide, and 2 mM potassium ferrocyanide in 50 mM sodium phosphate buffer (pH 7.0). After 15 min of vacuum infiltration, the samples were incubated overnight at 37 °C in the dark, followed by chlorophyll clearance with 70% ethanol. For ROS detection, duckweed and Arabidopsis seedlings were stained with either 0.1% DAB (3,3′-diaminobenzidine in 50 mM Tris-HCl, pH 5.0) or 0.1% NBT (nitroblue tetrazolium in phosphate buffer, pH 7.0) for up to 30 min, with the duration adjusted to achieve optimal staining intensity. Following staining, the seedlings were cleared in 95% ethanol for 1 h and mounted on glass slides using HCG solution (24 g chloral hydrate, 3 mL glycerol, 9 mL H_2_O). Imaging was performed using a Leica DM2500 microscope (Wetzlar, Germany). Image J software (v1.54r) was used to quantify the staining results.

### 2.3. Quantitative Real-Time PCR

Total RNA was extracted from 10-day-old Arabidopsis seedlings or roots using the TaKaRa MiniBEST Plant RNA Extraction Kit (TaKaRa, Osaka, Japan). First-strand cDNA was synthesized using the PrimeScript™ 1st Strand cDNA Synthesis Kit (TaKaRa, Osaka, Japan). Quantitative real-time PCR (qPCR) was performed on a QuantStudio 3 system (Thermo Fisher Scientific, Waltham, MA, USA) using the SYBR Premix Ex Taq™ II kit (TaKaRa, Osaka, Japan). Primer specificity was validated by melt curve analysis, and amplification efficiencies were determined using standard curves. Relative transcript levels were calculated using the comparative Ct method and normalized to the internal reference gene *ACTIN2*. All primer sequences are listed in Table S1.

### 2.4. ACC Quantification by ESI-HPLC-MS/MS

Plant tissue (100 mg) was extracted with 1 mL pre-cooled 80% methanol containing 0.1% formic acid and D4-ACC (internal standard). After ice-bath sonication for 30 min and centrifugation (12,000× *g*, 15 min, 4 °C), the supernatant was filtered (0.22 μm). LC-MS/MS analysis used a Waters BEH Amide column (2.1 × 100 mm, 1.7 μm; 40 °C) with a gradient of (A) 10 mM ammonium formate/0.1% formic acid in water and (B) 0.1% formic acid in acetonitrile (0–2 min, 90% B; 2–8 min, 90–50% B; 8–9 min, 50–90% B; 9–12 min, 90% B) at 0.3 mL/min. MS detection (SCIEX QTRAP 6500+) in positive ESI mode used MRM transitions: *m*/*z* 102.1→56.1 (ACC) and *m*/*z* 106.1→60.1 (D4-ACC). Method validation confirmed the linearity of the assay, with a recovery rate of 85.2–112.3%, a limit of detection (LOD) of 0.1 ng/mL, and a limit of quantification (LOQ) of 0.5 ng/mL. Absolute ACC content was calculated via isotope dilution.

## 3. Results

### 3.1. Sulfonamide-Induced Root Growth Inhibition Requires ACS1

**Figure 1:**
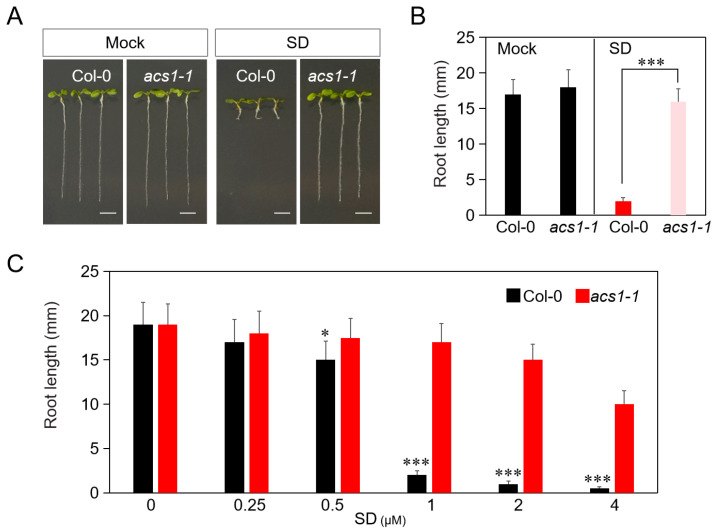
The *acs1-1* mutant is insensitive to sulfonamide. (**A**) Phenotypes of wild-type (Col-0) and *acs1-1* mutant Arabidopsis plants grown on 1/2MS medium with or without (Mock) 1 µM SD. Scale bar = 2 mm. (**B**) The root length of plants in A. Data were analyzed using Student’s *t*-test and are presented as the mean ± SE (*n* > 10). Asterisks indicate statistically significant differences (***, *p* < 0.01). (**C**) Dose–response effect of SD on root growth inhibition. For multiple comparisons, data were analyzed by Student’s *t*-test. Data are presented as the mean ± SE (*n* > 10). Asterisks indicate statistically significant differences (***, *p* < 0.01; *, *p* < 0.05).

Sulfonamide exposure severely impacts plant growth; even micromolar concentrations of sulfadiazine (SD) strongly inhibit root growth in the model plant *Arabidopsis thaliana* (Figure 1A). To investigate the underlying mechanisms, we utilized a mutant library of approximately 1500 key Arabidopsis mutants to perform a forward genetic screen on 1/2 MS medium containing 1 μM SD, identifying mutants with SD sensitivity significantly different from that of the wild-type Col-0 based on the root length of 7-day-old seedlings. Surprisingly, *acs1-1*, a loss-of-function mutant of the key ethylene biosynthetic enzyme ACS1, was identified as a highly insensitive allele (Figure 1A,B). Dose–response assays revealed that while 1 μM SD drastically inhibited wild-type (Col-0) root growth, *acs1-1* showed no phenotype until the concentration reached 4 μM (Figure 1C). Another loss-of-function mutant allele of *ACS1*, *acs1-2*, similarly displayed insensitivity to SD (Figure S1). These results indicate that SD-induced root growth inhibition strictly depends on ACS1.

### 3.2. Sulfonamide Exposure Strongly Induces ACS1 Expression

**Figure 2:**
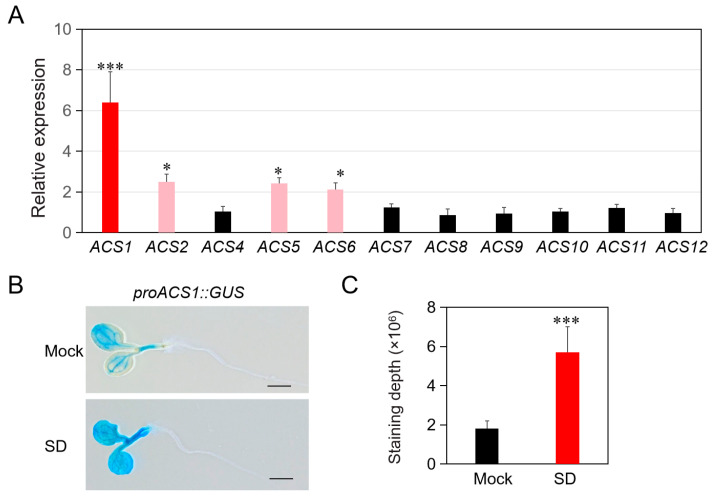
Sulfonamide exposure elevates ACS1 transcript levels. (**A**) qPCR analysis of ACS expression in the plants after 2 h of 1 µM SD treatment. The data represent three independent biological replicates. For multiple comparisons, data were analyzed by Student’s *t*-test. Asterisks indicate statistically significant differences (***, *p* < 0.01; *, *p* < 0.05). (**B**) GUS staining of proACS1-GUS plants treated after 2 h of 1 µM SD treatment. Scale bar = 2 mm. (**C**) Quantification of GUS staining intensity in (**B**). Image-based quantitative analysis was performed. Statistical assumptions of normality and homogeneity of variance were verified. Data were analyzed using Student’s *t*-test and are presented as the mean ± SE (*n* = 5). Asterisks indicate statistically significant differences (***, *p* < 0.01).

Given the complete insensitivity of the *acs1-1* mutant to SD, which suggests that SD-mediated root growth inhibition depends on ACS1, we investigated whether SD exposure induces *ACS1* expression. To test this, we used quantitative real-time PCR (qPCR) to analyze the expression profiles of *ACS1* and the other 10 *ACS* family members in Arabidopsis in response to SD. The results revealed that SD exposure for merely 2 h upregulated *ACS1* expression by approximately 5-fold, and *ACS2*, *ACS5* and *ACS6* showed about a 2-fold increase, and the remaining *ACS* genes exhibited no significant changes (Figure 2A). These findings demonstrate that SD exposure inhibits root growth specifically through the transcriptional induction of *ACSs*, especially *ACS1*.

To further corroborate the induction of *ACS1* by sulfonamide exposure, we generated transgenic plants expressing the *proACS1::GUS* reporter construct. Histochemical GUS staining revealed that sulfonamide exposure significantly enhanced both the spatial pattern and the intensity of GUS activity (Figure 2B,C). These results further reinforce the robust induction of *ACS1* in response to sulfonamides.

### 3.3. Sulfonamide Exposure Leads to a Surge in ACC Production

**Figure 3:**
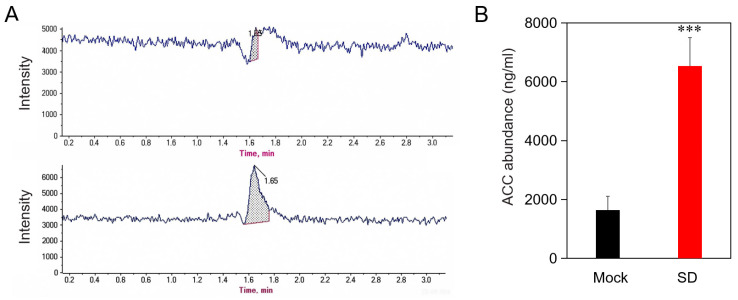
Sulfonamide elevates ACC abundance dramatically. (**A**) ESI-HPLC-MS/MS analysis of ACC levels in seedlings following 24 h exposure to 1 µM SD. (**B**) Quantification of ACC abundance. Statistical assumptions of normality and homogeneity of variance were verified. Data were analyzed using Student’s *t*-test. Three biological replicates were performed, and statistical significance is indicated by asterisks (***, *p* < 0.01).

Because sulfonamide-induced root growth inhibition is entirely dependent on ACS1—the rate-limiting enzyme in ethylene biosynthesis—we hypothesized that sulfonamide treatment triggers an ethylene burst in plants. To test this hypothesis, we measured the levels of the ethylene precursor ACC in Arabidopsis using ESI-HPLC-MS/MS after 24 h of sulfonamide exposure. The results showed that ACC accumulation in the sulfonamide-treated plants was more than 3-fold higher than that in the Mock controls (Figure 3). Taken together, we conclude that sulfonamide exposure specifically induces *ACS1* expression, leading to massive ACC accumulation, which subsequently drives excessive ethylene biosynthesis and ultimately inhibits root growth.

### 3.4. Inhibition of Ethylene Production Attenuates Sulfonamide-Mediated Root Growth Inhibition

**Figure 4:**
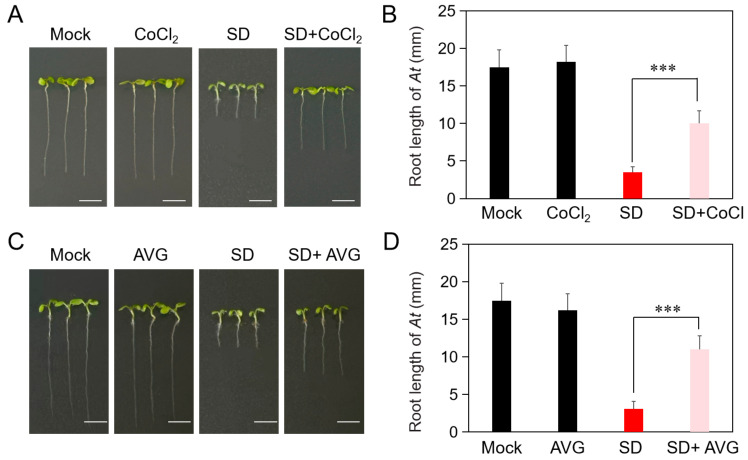
Ethylene biosynthesis inhibitors rescue sulfonamide-induced root growth inhibition. (**A**) Phenotypes of wild-type Arabidopsis seedlings grown on 1/2 MS medium under the treatments: Mock, 1 µM SD, 5 µM CoCl_2_ alone, or SD + CoCl_2_. Scale bar = 5 mm. (**B**) The root length of plants in (**A**). Data were analyzed using Student’s *t*-test and are presented as the mean ± SE (*n* > 10). (**C**) Phenotypes of wild-type Arabidopsis seedlings grown on 1/2 MS medium under the treatments: Mock, 1 µM SD, 5 µM AVG alone, or SD + AVG. Scale bar = 5 mm. (**D**) The root length of plants in C. Data were analyzed using Student’s *t*-test and are presented as the mean ± SE (*n* > 10). Asterisks indicate statistically significant differences (***, *p* < 0.01).

To further verify that sulfonamide-induced root growth inhibition relies on endogenous ethylene production, we co-treated plants with sulfonamide and either CoCl_2_ or AVG (aminoethoxyvinylglycine), both of which are classic ethylene biosynthesis inhibitors. The results showed that although CoCl_2_ and AVG failed to fully restore root length to normal levels, they significantly alleviated the inhibitory effect of sulfonamide on root growth (Figure 4). This evidence further corroborates that the inhibition of root growth by sulfonamide is primarily mediated by excess ethylene synthesis, and that blocking the ethylene biosynthetic pathway can effectively rescue this phenotype.

### 3.5. Sulfonamide Exposure Induces ACC Synthesis in a ROS-Dependent Manner

**Figure 5:**
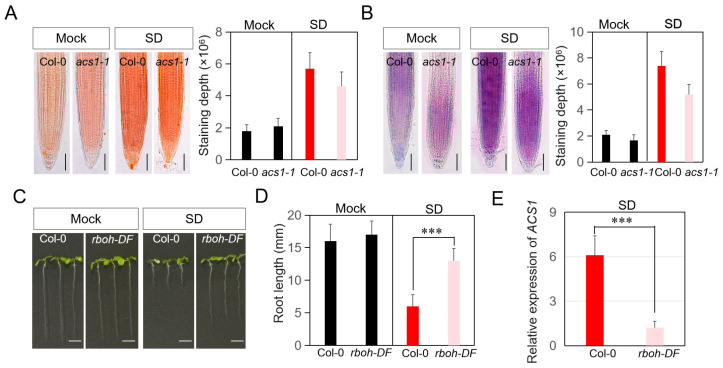
Sulfonamide-induced ethylene biosynthesis requires ROS. (**A**) DAB staining of H_2_O_2_ and (**B**) NBT staining of O_2_*·^−^* in Arabidopsis roots treated with 1 µM SD for 1 h. Image-based quantitative analysis was performed. Statistical assumptions of normality and homogeneity of variance were verified. Data were analyzed using Student’s *t*-test and presented as the mean ± SE (*n* > 5). Scale bar = 500 µm. (**C**) The *rboh-DF* mutant is partially insensitive to sulfonamide-induced root growth inhibition. Scale bar = 2 mm. (**D**) The root length of plants in C. Data were analyzed by Student’s *t*-test and presented as the mean ± SE (*n* > 10). Asterisks indicate statistically significant differences (***, *p* < 0.01). (**E**) qPCR analysis of *ACS1* expression in the *rboh-DF* mutant. Data were analyzed by Student’s *t*-test and presented as the mean ± SE. The data show one of three independent experiments. Asterisks indicate statistically significant differences (***, *p* < 0.01).

Under stress conditions, plants often experience bursts and abnormal accumulation of reactive oxygen species (ROS), which subsequently trigger stress responses. To determine whether sulfonamide exposure induces ROS accumulation in Arabidopsis, we performed DAB and NBT staining. The results revealed that sulfonamide treatment caused abnormal accumulation of hydrogen peroxide (H_2_O_2_) and superoxide anion (O_2_·^−^) in both wild-type and *acs1-1* mutants (Figure 5A,B). Respiratory burst oxidase homolog (RBOH), the primary NADPH oxidase responsible for ROS generation; its loss-of-function mutant, *rboh-DF*, exhibited partial resistance to sulfonamide exposure, indicating that abnormal ROS accumulation is a major cause of root growth inhibition (Figure 5C,D). To investigate whether ROS promotes ethylene biosynthesis by inducing *ACS1* expression, we examined *ACS1* transcript levels in the *rboh-DF* mutant under sulfonamide exposure using qPCR. Compared to the wild type, *rboh-DF* exhibited only 1/3 of the *ACS1* expression when treated with SD. Our findings indicate that the induction of *ACS1* by sulfonamides is mediated by ROS.

### 3.6. Sulfonamide Inhibits Root Growth Through Type 1 ACS

**Figure 6:**
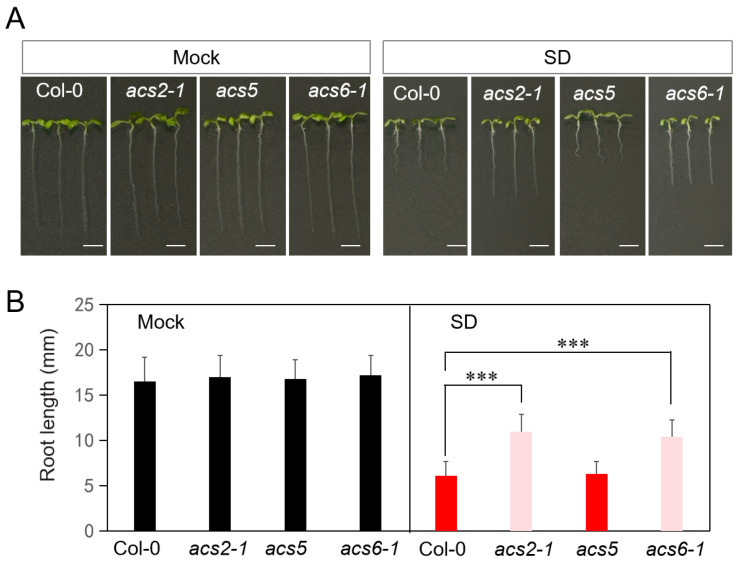
Sulfonamide targets Type 1 ACS. (**A**) The *acs2-1* and *acs6-1* mutants are partially insensitive to sulfonamide-induced root growth inhibition. Scale bar = 2 mm. (**B**)The root length of plants in (**A**). For multiple comparisons, data were analyzed by Student’s *t*-test and presented as the mean ± SE (*n* > 10). Asterisks indicate statistically significant differences (***, *p* < 0.01).

In Arabidopsis, ACS proteins are encoded by a 12-member gene family, but only eight are functionally active (ACS2, ACS4-9, and ACS11) [20]. The remaining genes encode inactive enzymes; specifically, ACS3 is a pseudogene, and ACS1 is catalytically inactive. Although ACS1 homodimers are nonfunctional, ACS1 can form active heterodimers with Type 1 members of its phylogenetic branch, notably ACS2 and ACS6 [21]. To this end, we analyzed the effects of sulfonamide exposure on the *acs2-1* and *acs6-1* mutants, finding that both exhibited partial insensitivity to sulfonamide-induced root growth inhibition (Figure 6). This result further demonstrates that sulfonamide exposure targets Type 1 ACS, leading to aberrant ACC synthesis in plants and thereby inhibiting root growth.

### 3.7. Sulfonamide Induces ACC Synthesis Partially via MPK3/6-Mediated Phosphorylation of ACS2/6

**Figure 7:**
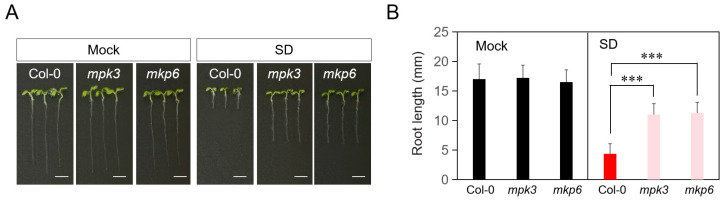
Sulfonamide triggers ACC via MPK3/6-phosphorylated ACS2/6. (**A**) The *mpk3* and *mpk6* mutants exhibit partial resistance to sulfonamide-mediated root growth inhibition. Scale bar = 2 mm. (**B**) The root length of plants in (**A**). For multiple comparisons, data were analyzed by Student’s *t*-test and presented as the mean ± SE (*n* > 10). Asterisks indicate statistically significant differences (***, *p* < 0.01).

Mitogen-activated protein kinases (MPKs) play crucial roles in regulating plant growth, development, and environmental responses [22]. Previous studies have demonstrated that MPK3/MPK6 promote ethylene biosynthesis by stabilizing ACS2/ACS6 via C-terminal phosphorylation [23]. To determine whether the aberrant ACC production induced by sulfonamide exposure depends on the MPK3/6 pathway, we examined the responses of *mpk3* and *mpk6* loss-of-function mutants to sulfonamide exposure. Both mutants displayed partial insensitivity, providing genetic evidence that sulfonamide-triggered ACC synthesis is partially mediated by the MPK3/6-ACS2/6 phosphorylation module (Figure 7).

## 4. Discussion

As a pleiotropic phytohormone, ethylene regulates diverse aspects of plant growth and stress adaptation, necessitating stringent spatiotemporal control over its biosynthesis [24,25]. ACS, the rate-limiting enzyme in this pathway, is subject to multi-layered regulation by environmental stresses [22,26]. Beyond transcriptional control, ACS activity is fine-tuned through heterodimerization; for instance, the catalytically inactive ACS1 can be rescued by forming active heterodimers with ACS2 or ACS6, relying on the latter to supply the essential active-site residue [20]. Aligning with this model, our genetic screening revealed a distinct hierarchy of sulfonamide sensitivity: the *acs1-1* mutant exhibited insensitivity, whereas *acs2-1* and *acs6-1* were only partially insensitive. We demonstrate that sulfonamide exposure coordinately upregulates the expression of *ACS1*, *ACS2*, and *ACS6*. This induction raises the possibility that sulfonamide stress may drive the formation of both ACS homodimers and ACS1-containing heterodimers, which could cooperatively trigger an aberrant ethylene burst and subsequent root growth inhibition.

The integration of environmental stress cues into cellular responses relies heavily on reactive oxygen species (ROS) [27,28]. While ROS homeostasis is strictly maintained under steady-state conditions, stress rapidly disrupts metabolic pathways, prompting ROS accumulation in compartments such as the apoplast and chloroplasts [29]. Notably, RBOH-driven apoplastic ROS production acts as a pivotal early signaling hub, propagating stress signals from the cell periphery to the nucleus [30,31]. Our findings position this RBOH-dependent ROS burst directly downstream of sulfonamide toxicity. Sulfonamide induces aberrant ROS accumulation, and the *rboh-DF* mutant displays partial insensitivity to sulfonamide-induced growth inhibition, underscoring the mediating role of ROS. Crucially, the drastic attenuation of sulfonamide-induced *ACS1* expression in the *rboh-DF* background reveals that ROS functions as an indispensable signaling intermediate, potentially linking sulfonamide perception to the activation of ethylene biosynthesis via *ACS1*.

To translate these early ROS signals into hormonal responses, plants employ conserved signaling cascades [32]. Upon stress perception, early events such as calcium influx and protein phosphorylation rapidly trigger the generation of signaling molecules and phytohormones, including ethylene [32,33,34]. Mitogen-activated protein kinase (MPK) cascades serve as major conduits for transducing extracellular stimuli into intracellular responses [22]. In Arabidopsis, stress-responsive MPK3 and MPK6 directly promote ethylene biosynthesis by phosphorylating ACS isoforms [30,35]. Specifically, C-terminal phosphorylation of ACS2/ACS6 by MPK3/MPK6 stabilizes these enzymes and enhances their catalytic activity, thereby driving ethylene production [36]. The partial insensitivity of *mpk3* and *mpk6* mutants to sulfonamide provides genetic evidence that the MPK3/MPK6 pathway functions downstream of sulfonamide stress. Taken together with our findings on the ROS-*ACS* axis, we propose that sulfonamide stress is decoded through a bifurcated signaling network: one branch relies on the RBOH-dependent ROS burst to specifically activate *ACS1* transcription, while the other utilizes the MPK3/MPK6 cascade to phosphorylate and stabilize ACS2/ACS6. These signaling branches converge on the ethylene biosynthesis machinery, culminating in excessive ethylene accumulation and the ultimate suppression of root growth.

## 5. Conclusions

Sulfonamides inhibit root growth by perturbing the ROS-ACC synthesis pathway. SD-induced ROS bursts transcriptionally activate ACS1, and genetic evidence implicates MPK3/6 in the partial post-translational stabilization of ACS2 and ACS6. The convergence of these mechanisms drives excessive ACC accumulation, culminating in phytotoxicity.

## Data Availability

The original contributions presented in this study are included in the article. Further inquiries can be directed to the corresponding author.

## Supplementary Materials

The following supporting information can be downloaded at https://www.mdpi.com/article/10.3390/biology15151278/s1. Figure S1: The *acs1-2* mutant is insensitive to sulfonamide; (**A**) phenotypes of wild-type (Col-0) and *acs1-2* mutant Arabidopsis plants grown on 1/2MS medium with or without (Mock) 1 µM SD. Scale bar = 2 mm. (**B**) The root length of plants in (**A**). Data are presented as the mean ± SE (*n* > 10). Asterisks indicate statistically significant differences (***, *p* < 0.01); Table S1: Primers used in the study; Table S2: Primers AGI code of genes.

## Abbreviations

The following abbreviations are used in this manuscript:

SD	Sulfadiazine
ROS	Reactive oxygen species
ACC	1-aminocyclopropane-1-carboxylate
ACS	ACC synthase
DAB	3,3′-diaminobenzidine
NBT	Nitroblue tetrazolium
MPKs	Mitogen-activated protein kinases
GUS	β-glucuronidase
ARGs	Antibiotic resistance genes
