# Supplementary material for: Sulfonamides Inhibit Root Growth via ROS-Triggered and MPK3/6-Modulated Synthesis of the Ethylene Precursor ACC
Source: Biology (Basel). 2026 Aug 3;15(15):1278. doi: 10.3390/biology15151278 (PMC13464784; doi:10.3390/biology15151278)
Supplement: Supplementary file 1 [file biology-15-01278-s001.zip › biology-4388653-supplementary.pdf]

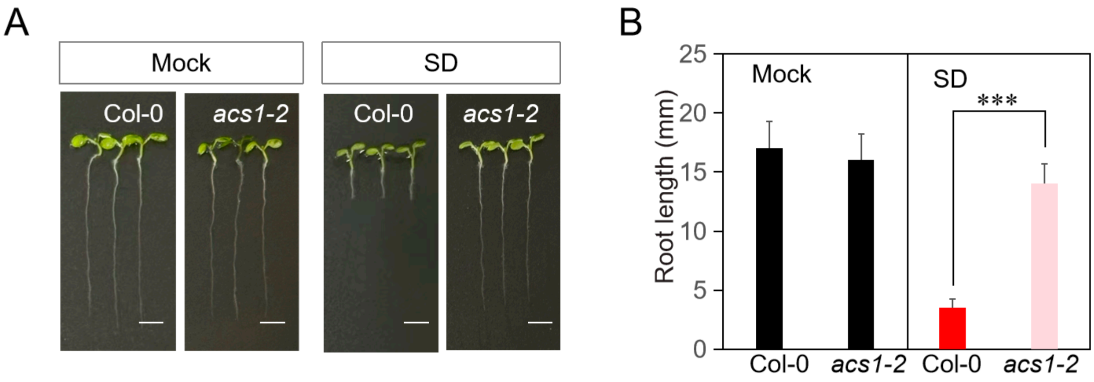

Figure S1. The *acs1-2* mutant is insensitive to sulfonamide. (**A**) Phenotypes of wild-type (Col-0) and *acs1-2* mutant Arabidopsis plants grown on 1/2MS medium with or without (Mock) 1 μM SD. Scale bar = 2 mm. (**B**) The root length of plants in A. Data are presented as the mean ± SE (n > 10). Asterisks indicate statistically significant differences (***, p < 0.01).

Table S1. Primers used in the study.

Name	Sequences	Process
ACS1-F	ACTCTTTCTCCGACATTG	Real-time qPCR
ACS1-R	AACTCCAGTTCTCCACCT	Real-time qPCR
ACS2-F	AAGCCCAAGAGTCCAATA	Real-time qPCR
ACS2-R	TCTTCCTCGTGACAAACC	Real-time qPCR
ACS4-F	CCAGGGTTTGATAGGGAT	Real-time qPCR
ACS4-R	TAGGCTTCTTCGAGTGCA	Real-time qPCR
ACS5-F	CCAAATCACGGAATCAGC	Real-time qPCR
ACS5-R	GCAGTGCCAAGTGGGTTA	Real-time qPCR
ACS6-F	TTTCGGTCTTGTTTCGTC	Real-time qPCR
ACS6-R	TGGTTATCTCAGCGTGCC	Real-time qPCR
ACS7-F	AGATTCGAGGAGGCAAAG	Real-time qPCR
ACS7-R	TAGAAGGGCGTCGTTAGG	Real-time qPCR
ACS8-F	TTGGGCACTACAACGACA	Real-time qPCR
ACS8-R	TGAGGACTTCCATTACGC	Real-time qPCR
ACS9-F	TGAAATGGAGAACGGGAGC	Real-time qPCR
ACS9-R	TGCCAAGAGGGTTAGACG	Real-time qPCR
ACS10-F	CGGATATGATAGGGATGT	Real-time qPCR
ACS10-R	GCTTGATAGAATGCTCGA	Real-time qPCR
ACS11-F	GGGTACAAGATAACCGAGGAT	Real-time qPCR
ACS11-R	CTTCACGGGTGGTAGAGG	Real-time qPCR
ACS12-F	CATACTATCAAGGGAGACG	Real-time qPCR
ACS12-R	CAGCCATGCTAACAAACT	Real-time qPCR
ACTIN2-F	GCTGACCGTATGAGCAAAGA	Real-time qPCR
ACTIN2-R	GATCCACATCTGTTGGAACG	Real-time qPCR

Table S2. Primers AGI code of genes

Name	AGI code
ACS1	AT3G46630
ACS2	AT1G01480
ACS3	AT2G17990
ACS4	AT2G22810
ACS5	AT5G65800
ACS6	AT4G11280
ACS7	AT4G26200
ACS8	AT4G37770
ACS9	AT3G49700
ACS10	AT1G62960
ACS11	AT4G08040
ACS12	AT5G51690
RBOH-D	AT5G47910
RBOH-F	AT1G34040
MPK3	AT3G45640
MPK6	AT2G43790
